# Analysis of goal scoring patterns in the UEFA Women’s EURO 2022

**DOI:** 10.5114/biolsport.2025.142646

**Published:** 2024-08-30

**Authors:** Jorge Sanmiguel Codina, Rafael Ballester Lengua, Claudio A. Casal, Florentino Huertas Olmedo

**Affiliations:** 1 Doctoral School, Catholic University of Valencia “San Vicente Mártir”, 46008 Valencia, Spain; 2 Faculty of Physical Education & Sport Sciences, Catholic University of Valencia “San Vicente Mártir”, 46900, Torrent, Valencia, Spain

**Keywords:** Football, Soccer, Technical-tactical performance, Women’s football, Goal scoring, Footballperformance analysis, Tactical analysis

## Abstract

Despite the growing interest in research on women’s football, few approaches have analysed the primary performance indicator in football, which is the goal. This study describes the goal scoring patterns and explores technical-tactical behaviours associated with the scorer location in the 2022 UEFA Women’s European Championship. The sample was made up of all the 95 goals scored by the 16 teams in the 31 games played. Three UEFA Pro coaches designed an ad hoc observational instrument, and one observer coded the data after a training process, ensuring intraobserver reliability. Descriptive and inferential analyses were conducted. Assistant location was significantly associated with scorer location (p < 0.005, ES = 0.930). The highest percentage of assists came from the lateral corridors outside the penalty area (23.2%). The most frequent shot zones being the projection from the goal line to the penalty spot (48.2%). It is also remarkable that half of the goals came from an aerial ball (51.2%), the leg (66.3%) and a first contact shot (69.8%) being the most common finishing situations. A similar distribution was noted concerning the areas where shots crossed the goal line (40.0% near post vs 41.3% far post). Our findings reveal goal-scoring patterns in elite women’s football to consider in the coaching process, for instance, to design drills to promote the replication of the most common finishing situations (aerial, leg and first contact) and contexts where most recurrent assisting and shooting zones could be considered. Raising awareness among coaches and players about these practical aspects can improve goal-scoring situations.

## INTRODUCTION

In recent years women’s football has experienced exponential growth leading players from the top leagues in many countries to attain professional status [1]. This development, together with the growing interest of researchers in creating a robust knowledge base, is contributing to improving performance in women’s football [2]. However, compared to men’s football, studies in this area are still very limited, accounting for only 20% of the total publications on football, with particular emphasis on research regarding sports injuries, physical requirements, and conditioning training [3, 4]. In this context, it is crucial to investigate the impact of individual and collective technical-tactical indicators on performance, particularly concerning the creation of goal-scoring opportunities. This study specifically addresses this objective within the elite framework of the UEFA Women’s Euro 2022.

The study of goals, as a crucial criterion of performance in football, has been a recurring topic among researchers [5]. However, most of this previous investigation has focused on the analysis of men’s football or in the comparison of different game indicators between genders. Preceding studies addressing the shooting situations found that the average number of shots on goal per match and the zones where the goals were achieved were similar between men and women [6, 7, 8]. In contrast, other authors reported that in women’s football there were more attempts made towards the opposing goal [9] and the average number of goals scored per match was higher than in men’s football [7, 8, 10, 11]. In men’s football, shots taken from greater distances have been noted [9, 12], whereas in women’s football, more goals have been scored with headers and shots with a lower angle with respect to the goal [9].

Regarding other technical-tactical aspects linked to the attacking situations, Mitrotasios et al. [13] observed that men’s teams had longer possessions, with a greater number and higher speed and accuracy of passes than their women’s counterpart [6, 14, 15]. Focusing on the defensive phase, Casal et al. [6] reported a greater number of interceptions and defensive recoveries by women’s teams in the opposite field. Similarly, Pappalardo et al. [12] highlighted that women’s football exhibited a lower level of tactical-strategic control compared to men’s football, which resulted in a greater number of duels, recoveries and turnovers.

Previous research on the analyses of technical-tactical differences between football of both genders in the attacking phase, especially in shooting situations, has certain limitations associated with transferring knowledge obtained from men’s football to the women’s game [3, 6, 15]. Thus, given the scarcity of studies and the diversity of methodological approaches applied in previous research, there is a need of further investigation focusing specifically on the variables that influence goal achievement in women’s football.

One of the most recurrent topics in research related to goal scoring in women’s football is the analysis of the timing of goals during the match and their impact on the final outcome. Armatas et al. [16] found that in the 1995, 1999, and 2003 Women’s World Cups, the majority of goals were scored in the second half, with an increasing trend in the number of goals as the match progressed. Other studies [17, 18] replicated these findings in both the London 2012 Olympic Games and the Spanish First Division, confirming the trend (a higher number of goals between minutes 61 and 75 and the last quarter of the match). However, another study based on three elite European teams showed that more goals were scored near halftime [19]. Previously, Ibáñez et al. [20] investigated the impact of the first goal on the final result of the match in the Spanish First Division, discovering that 40.9% of the first goals were scored in the first 15 minutes of play, the better-ranked teams being more likely to score first and win the match. A similar pattern of results was described in the 2019 Women’s World Cup [21, 22]. Therefore, although scoring first in women’s football can bring a team closer to victory, it is important to consider that goals may come in the second half, as it is the most productive period according to the majority of research.

Espada et al. [23] compared goal-predicting variables between genders in the Portuguese league, finding some differences associated to gender in the actions preceding the goal (recovery zones, last-pass zones, shooting zones, and body zones used for shooting). Subsequently, Wang & Qin [22] conducted a descriptive analysis of goal-scoring patterns in women’s football at the 2019 World Cup, confirming that two-thirds of the goals originated from dynamic attack situations (with the remaining third coming from set-pieces) and that 79% of the goals were achieved through sequences of 0–4 passes. The results also showed that nearly half of the goals from dynamic attacks were scored with the first touch, and the central zone between the penalty spot and the goal area was the most frequent zone for shooting, aligning with previous studies in European football teams [18, 19].

When considering the generalization and transferability of the results from previous studies, it is crucial to recognize certain limitations that may arise from the sample size, the diversity of competitive contexts analysed, and the period in which each championship was held, taking into account the exponential growth of women’s football in recent years. Our literature review underscores the limited number of studies delving deeply into the analysis of goal-scoring actions, particularly considering relevant technical-tactical indicators associated with assists and goal shots in elite women’s football. Specifically, our study analyses previously unexplored technical-tactical indicators, such as the relationship between the assist and shot zone, the ball disposition during the assist or the shot direction on goal. This information can help to better identify and contextualise the most common finishing patterns for their consideration during the coaching process and game plan preparation.

## MATERIALS AND METHODS

### Design and sample

All the goals (n = 95) scored by the 16 teams participating in the 31 matches of the final stage (first round, quarter-finals, semi-finals and final) of the UEFA Women’s EURO 2022 were analysed postevent and extracted from the match reports provided by the WyScout database (Wyscout Spa, Italy) (see Wyscout documentation for further details at https://apidocs.wyscout.com/) and InStat (currently out of service). Many of the most relevant studies on this topic cited in our research have used both Wyscout [12, 13, 19, 23] and InStat [6, 24].

The unit of analysis spanned from the last ball contact by the goal assister until the moment the ball entered the goal. The information was recorded by observing the behaviour of the players on the playing field through a post-event record. According to the Belmont Report [25], the use of public images for research purposes does not require informed consent or the approval of an ethical committee.

### Observation instrument

The observation instrument has been developed *ad hoc*, being a combination of a field format and systems of categories [26], taking into account various studies in the field [2, 6, 13, 22, 23]. This process was carried out by four expert researchers (three having PhDs in Sports Sciences, two of them football coaches with a UEFA Pro license) with more than ten years of training and research experience on this topic. After reviewing the previous empirical evidence mentioned throughout the present study, the researchers developed the observation instrument. Next, a first exploratory post-event observation of all the goals was carried out by one researcher to verify whether the description of the categories fit all the actions studied. In this stage, those situations that could not be addressed or that could be simplified into fewer categories were noted on a case-bycase basis. Subsequently, and after a discussion by the whole team of experts, the observation instrument was readjusted, delimitating more accurately the categories of each criterion. Afterwards, the *ad hoc* observation instrument was tested again by the research team in those events that had been defined as “controversial” in addressing one or other category. This procedure was repeated until the researchers reached a consensus on the criteria for categorizing those contentious actions, ensuring that the observation and categorization of the event were unequivocal. Finally, the final post-event viewing was carried out (see Table 1).

**TABLE 1:** Criteria, definition and codes of the observation instrument.

Criteria	Definition	Categories	Code
Time	Timing of the goal within the match (Wyscout*)	**Between minutes 0–15**	0–15
		**Between minutes 16–30**	16–30
		**Between minutes 31–45+** (until the end of the 1^st^ Half)	31–45+
		**Between minutes 45–60** (since the start of the 2^nd^ Half)	45–60
		**Between minutes 61–75**	61–75
		**Between minutes 76–90+** (until the end of the match)	76–90+
		**Overtime (OT):** in quarterfinals, semifinals and final	OT
Impact of the first goal	Influence of the first goal scored in the match on the eventual outcome	**No goals**	NG
		**Wins**	W
		**Draws**	D
		**Loses**	L
Attack type	Specific method of attack employed by the team during the play that leads to the goal (InStat**)	**Dynamic attack:** The goal is scored as a result of a positional attack or counterattack	DA
		**Set-piece:** The goal is scored from a set-piece situation, including corners, free kicks in favor, direct free kicks, throw-ins, and penalties	SP
Assistant location	Positioning of the assisting player on the field at the moment of the pass. Penalties and direct free kicks are excluded (Figure 1)	**Attacker:** The final touch on the ball before it reaches the scorer is made by an attacking player	AZ1-AZ21
		**Defender:** The final touch on the ball before it reaches the scorer is made by a defending player
Scorer location	Position on the field where the player who scores the goal is situated at the moment of the shot. Penalties are excluded (Figure 2)	**Voluntary:** the goal is scored by an attacking player	GZ1-GZ13
		**Involuntary:** the goal is scored by a defending player (goal on own goal)
Ball disposition	Manner in which the ball arrives to the shooter. Penalties and direct free throws are excluded	**Ground:** the ball reaches the shooter at ground level	GR
		**Aerial:** the ball reaches the shooter without making contact with the ground	AR
		**Bounce:** the ball reaches the shooter after the ball bounces	B
Number of scorer contacts	Count of the ball touches by the scorer during the finishing action. Penalties and direct free kicks are excluded	**One contact**	1C
		**Two contacts**	2C
		**Three or more contacts**	3C+
Contact surface at the shot	Body surface utilized by the scorer to execute the shot and score the goal. Penalties and direct free kicks are excluded	**Leg**	L
		**Head**	H
		**Others**	O
Shot distance	Distance between the ball and the goal at the moment of the shot. Penalties are excluded (InStat**)	**Short:** 0–5.5 meters (from the finish line to the small area)	S
		**Medium:** 5.6–11 meters (from the small area to the penalty spot)	M
		**Long:** 11.1–16.5 meters (from the penalty spot to the large area)	L
		**Very long:**> 16.6 meters (outside the large area)	VL
Scoring opposition	Presence of an opponent actively pressing the goal scorer at the moment of the shot, with the intention of obstructing the scoring action. Penalties, direct free kicks and goals on own goal are excluded	**With opposition:** Presence of one or more opposing players actively attempting to prevent the scoring action by the scorer, excluding the goalkeeper	OP
		**Without opposition:** Absence of any opposing players actively attempting to prevent the scoring action by the scorer, excluding the goalkeeper	NOP
Goal penetration zone	Specific area within the frontal plane of the goal where the ball enters to score a goal (InStat**) (Figure 3)	**Zone 1 to zone 6**	PZ1-PZ6
Shot direction	Direction from which shots enter the goal, categorized as either first post, second post, or center. Penalties, goals on own goal and goals from outside the area are excluded (See Fig. 3)	**From right side of the area:** Right half of the penalty area, delineated by a vertical line extending from the center of the goal to the penalty spot, with reference to the direction of the attack.	RS (1P, 2P o CT),
		**From right side of the area:** Left half of the penalty area, delineated by a vertical line extending from the center of the goal to the penalty spot, with reference to the direction of the attack	LS (1P, 2P o CP)

Note:

* Wyscout provides the glossary of criteria at the following link: https://dataglossary.wyscout.com/

** InStat’s database is no longer operational, so the glossary of criteria cannot be accessed.

### Procedure and reliability analysis

Data were coded post-event by one observer. The intra-observer reliability of the data was calculated through Cohen’s kappa [27] for each category, re-analysing the data from 30 randomly selected goals (> 10%, [28]) with a latency period of one month. The obtained agreement values (0.97) were interpreted as “almost perfect” according to the Fleiss index [29], as the kappa index values ranged from 0.89 to 1.00.

### Statistical analysis

In accordance with the aims of the study, both descriptive (frequency distribution tables) and inferential statistics (bivariate analysis) were used. The bivariate analyses were carried out by means of Pearson’s χ^2^ and the association between the “scorer location” and the technical-tactical explanatory variables was examined. In the inferential analysis, the “attack type” was excluded as only goals obtained in dynamic attacks were considered. The “shot distance” and “shot direction” were also excluded as they provided similar information to the “scorer location” and “goal penetration zone”. To mitigate the effect of chance, “involuntary” actions (assisted by a defending player or goals on own goal) were also excluded. Effect size was calculated from the contingency coefficient and described as small (ES = 0.10), medium (ES = 0.30) or large (ES > 0.50) [30]. To check if the observed values correspond to the expected ones in each cell of the contingency table analysed, the adjusted residual test was applied. To do this, the existence of possible differences was contrasted with a reference probability lower than 5% (Z > 1.96 or Z < -1.96) to assume such differences, considering a normal frequency distribution. Following this significance level, in cases where the difference for each criterion (observed versus expected in each cell) was greater than expected, the adjusted residual was considered positive, and negative in the opposite case. SPSS Statistics software Version 27.0 (IBM Corp., Armonk, NY) was used to conduct all the analysis, and the significance level for each category was set at 5%.

## RESULTS

### Descriptive analysis

In the championship, 95 goals were scored in 31 matches (3.1 goals per game), with only one match ending goalless. Out of the goals scored, 58 (61.0%) were scored in dynamic attacking situations, and 37 (39.0%) came from set-piece actions: 8 penalty (8.4%), one direct free kick (1.1%), 9 from free kicks (9.5%), 18 corner kick (18.9%), and one throw-in (1.1%).

### Contextual variables

The majority of goals were scored in the last fifteen minutes of each half of the match (24.2% and 21.1% respectively). Three matches went into overtime, with one goal scored in each (3.2% of the total goals). No match was decided by a penalty shootout.

Regarding the relevance of scoring the first goal and its impact on the final result, it is worth noting that only one team managed to come back after conceding the first goal of the match to ultimately win it at the end of regular time (Table 2).

**TABLE 2:** Absolute and relative frequencies of the categories of the observation instrument: *contextual variables*.

Time	Frecuency	%
0–15	14	14.7
16–30	8	8.4
31–45+	23	24.2
45–60	17	17.9
61–75	10	10.5
76–90+	20	21.1
OT	3	3.2
Total	95	100
		
**Impact of the first goal**	**Frecuency**	**%**
No goals	1	3.2
Wins	22	71.1
Draws	7	22.6
Loses	1	3.2
Total	31	100

### Technical-tactical indicators

In the analysis of the technical-tactical indicators that influenced goal assists, 86 assists were considered, achieved by either an attacking player (n = 68) or a defender through a rebound or involuntary action (n = 18). The analysis did not include the 8 penalty goals and 1 goal achieved from a direct free kick.

The primary zones for creating assists were Z1 and Z8, accounting for 23.2% of the total assists (n = 20). Specifically, in Z1, there were 6 assists from dynamic attacks, 1 from a free kick in favour, and 4 from corner kicks. Meanwhile, Z8 contributed with 4 assists from dynamic attacks and 5 from corner kicks. This record is similar to those obtained in z7a+z7b and z2a+z2b (with an assistance zone dimension comparable to that of z8 and z1), which were also executed by an attacking player. However, in neither case did the ball come directly from a set piece shot.

**FIG. 1:**
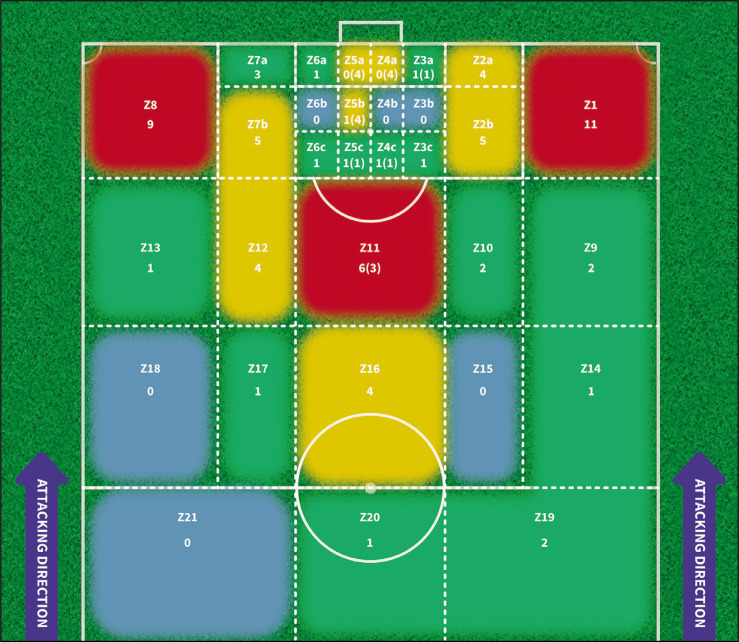
Distribution of frequency of goals according to the Assistant Location. Note: The table displays the frequency of assists, where the final touch on the ball before it reaches the scorer comes either an attacker (n = 68) or a defender (n = 18; in parentheses). Direct free kicks (n = 1) and penalties (n = 8) were excluded. The colors correspond to frequency intervals for assists: in red for 11–8 assists; in yellow for 7–4 assists; in green for 3–1 assists; in blue for areas where no assists have been performed.

Eighteen of the assists (20.9%) were assists resulting from involuntary actions by a defending player (loss or rebound), with the majority of them (n = 12) located in the zones between the goal line and the penalty spot (see Figure 1 and Table 3).

**TABLE 3:** Absolute and relative frequencies of the categories from the observation instrument: *technical-tactical indicators*.

Criteria & Category/Code	Frecuency	%
*Type of attack*		
*Set-piece actions (SP)*	37	39.0
Corner	18	18.9
Free kicks in favor	9	9.5
Direct free kicks	1	1.1
Throw-ins in favor	1	1.1
Penaltis	8	8.4
*Dynamic attack (DA)*	58	61.0
Total	95	100
*Assistant location (AZ)*		
*Attacker*	68	79.1
Zone 1	11	12.8
Zone 2a	4	4.6
Zone 2b	5	5.8
Zone 3a	1	1.2
Zone 3c	1	1.2
Zone 4c	1	1.2
Zone 5b	1	1.2
Zone 5c	1	1.2
Zone 6a	1	1.2
Zone 6c	1	1.2
Zone 7a	3	3.5
Zone 7b	5	5.8
Zone 8	9	10.4
Zone 9	2	2.3
Zone 10	2	2.3
Zone 11	6	6.9
Zone 12	4	4.6
Zone 13	1	1.2
Zone 14	1	1.2
Zone 16	4	4.6
Zone 17	1	1.2
Zone 19	2	2.3
Zone 20	1	1.2
*Defender*	18	20.9
Zone 3a	1	1.2
Zone 4a	4	4.6
Zone 4c	1	1.2
Zone 5a	4	4.6
Zone 5b	4	4.6
Zone 5c	1	1.2
Zone 11	3	3.5
Total	86	100
*Scorer location (GZ)*		
*Voluntary*	83	95.5
Zone 2b	1	1.1
Zone 3a	6	7.0
Zone 3b	6	7.0
Zone 3c	3	3.5
Zone 4a	12	13.8
Zone 4b	13	14.9
Zone 4c	3	3.5
Zone 5a	8	9.2
Zone 5b	9	10.3
Zone 5c	6	7.0
Zone 6a	1	1.1
Zone 6b	2	2.3
Zone 6c	3	3.5
Zone 7a	1	1.1
Zone 7b	1	1.1
Zone 10	1	1.1
Zone 11	5	5.7
Zone 12	2	2.3
*Involuntary*	4	*Involuntary*
Zone 4a	1	Zone 4a
Zone 4c	1	Zone 4c
Zone 5a	2	Zone 5a
Total	87	Total
*Ball disposition*		
Ground (GR)	24	27.9
Aerial (AR)	44	51.2
Bounce (B)	18	20.9
Total	86	100
*Nº of scorer contacts*		
One contact (1C)	60	69.8
Two contacts (2C)	16	18.6
Three + contacts (3C+)	10	11.6
Total	86	100
*Contact surface at the shot*		
Leg (L)	57	66.3
Head (H)	28	32.5
Others (O)	1	1.2
Total	86	100
*Shot distance*		
Short (S)	21	24.1
Medium (M)	35	40.2
Long (L)	22	25.3
Very long (VL)	9	10.4
Total	87	100
*Scoring opposition*		
With opposition (OP)	40	48.8
Without opposition (NOP)	42	51.2
Total	82	100
*Goal penetration zone*		
Zone 1 (PZ1)	8	8.4
Zone 2 (PZ2)	30	31.6
Zone 3 (PZ3)	7	7.4
Zone 4 (PZ4)	11	11.6
Zone 5 (PZ5)	13	13.7
Zone 6 (PZ6)	26	27.3
Total	95	100
*Shot direction*		
Right profile (RD)	44	58.7
First post (1P)	17	22.7
Second post (2P)	16	21.3
Center (CT)	11	14.7
Left profile (LD)	31	41.3
First post (1P)	13	17.3
Second post (2P)	15	20.0
Center (CT)	3	4.0
Total	75	100

**FIG. 2:**
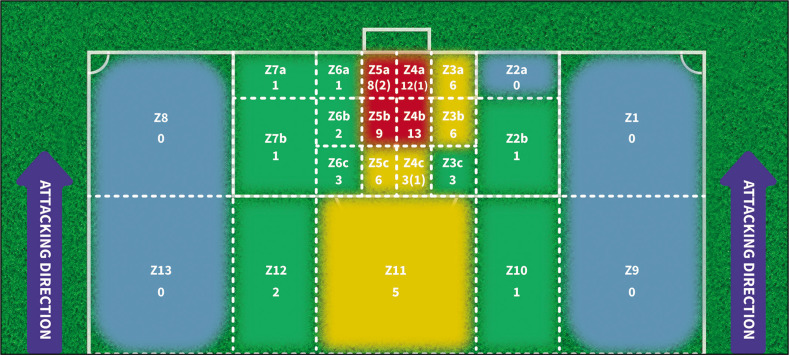
Distribution of frequency of goals according to the Scorer Location. Note: The table displays the frequency of goals which come voluntarily (n = 83) or involuntarily (n = 4; in parentheses). Goals scored from penalty kicks (n = 8) were excluded. The colors correspond to intervals of frequency for goals: in red for 13–9 goals; in yellow for 8–4 goals; in green for 3–1 goals; in blue for zones where no goals have been scored.

In relation to the scorer’s location, the 8 goals scored from penalty kicks were excluded from the analysis. Our results show that out of the remaining 87 goals (83 intentional and 4 unintentional), 42 of them (48.2%) were scored voluntarily from the zones that extends from the goal line to the penalty spot. Additionally, 8 goals (9.1%) were scored from the front of the penalty area (Z10, Z11, and Z12) (see Figure 2 and Table 3).

Regarding the ball disposition, in 44 instances (51.2%), the ball was received by the scorer without touching the ground after the assist (ball aerial). In most goal shots (69.8%), the shot was taken with a first touch. Regarding the body surface used in the shot, 57 goals were scored with the legs (66.3%) and 28 with the head (32.5%).

Concerning shot distance from the goal, the most frequent range was 5.6–11 m (ZB) with 35 goals (40.2%). Additionally, in 42 instances (51.2%), there was no opposition from a defending player during the shot.

**FIG. 3:**
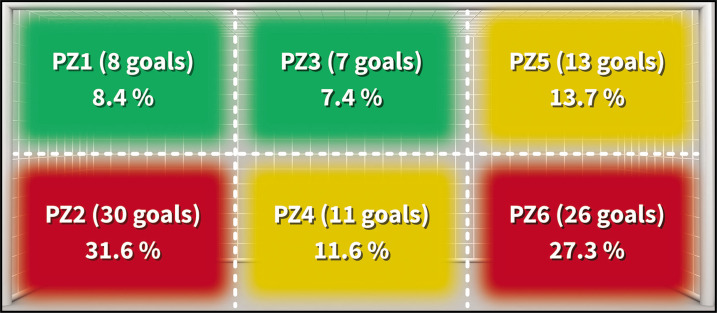
Distribution of frequency of goals according to the Goal Penetration Zone. Note: The colors correspond to intervals of frequency for the goal penetration zone (n = 95): in red for 30–20 goals; in yellow for 19–10 goals; in green for 9–1 goals.

In terms of the goal zone where the goals entered (goal penetration zone, PZ), considering a frontal plane of the goal divided into 6 zones (PZ), it was found that the majority of goals (56, equivalent to 58.9% of the total) were scored in the low angles (PZ2 and PZ6) (Figure 3).

A very similar distribution was observed in the goals scored from inside the penalty area, whether they entered at the near or far post (Table 3), regardless of the shot direction (based on the attacking direction and excluding the 8 penalty goals, 4 own goals, and 8 goals from outside the penalty area). It was found that out of the 44 intentional shots from the right profile inside the area (Figure 2; from Z2a to Z4c), 17 balls (22.7%) entered at the near post (PZ5 and ZP6), 11 (14.7%) through the centre (PZ3 and PZ4), and 16 (21.3%) at the far post (PZ1 and PZ2). On the other hand, out of the 31 intentional shots from the left profile (Figure 2; from Z5a to Z7b), 13 goals (17.3%) entered at the near post (PZ1 and PZ2), 3 (4.0%) through the centre (PZ3 and PZ4), and 15 (20.0%) at the far post (PZ5 and PZ6).

### Bivariate analysis

Table 4 displays the criteria and categories (technical-tactical indicators, except for “attack type”, “shot distance”, and “shot direction”) significantly associated (*p* < 0.05) with the scorer’s location. In the analysis, both set-pieces and “involuntary” plays (assistant or scorer from the opposite team) were excluded, with the resultant sample being n = 44.

**TABLE 4:** Count and relative percentage of goals scored according to the scorer location and technical-tactical indicators in dynamic attacks.

Criteria	Scorer location															χ^2^ value p value Cont. Coef.

Assistant location	GZ2b	GZ3a	GZ3b	GZ3c	GZ4a	GZ4b	GZ5a	GZ5b	GZ5c	GZ6b	GZ6c	GZ7b	GZ10	GZ11	GZ12	
AZ1	0/0%	0/0%	1/33.3%	0/0%	0/0%	3/33.4%	1/20%	1/16.7%	0/0%	0/0%	0/0%	0/0%	0/0%	0/0%	0/0%	283.930 0.004 0.930

AZ2a	0/0%	0/0%	0/0%	0/0%	0/0%	0/0%	2+*/40%	0/0%	0/0%	0/0%	0/0%	0/0%	0/0%	0/0%	0/0%	

AZ2b	0/0%	0/0%	1/33.3%	0/0%	1/25%	1/11.1%	1/20%	1/16.7%	0/0%	0/0%	0/0%	0/0%	0/0%	0/0%	0/0%	

AZ3a	0/0%	0/0%	0/0%	0/0%	0/0%	0/0%	1+*/20%	0/0%	0/0%	0/0%	0/0%	0/0%	0/0%	0/0%	0/0%	

AZ5b	0/0%	0/0%	0/0%	0/0%	1+*/25%	0/0%	0/0%	0/0%	0/0%	0/0%	0/0%	0/0%	0/0%	0/0%	0/0%	

AZ6a	0/0%	0/0%	0/0%	0/0%	1+*/25%	0/0%	0/0%	0/0%	0/0%	0/0%	0/0%	0/0%	0/0%	0/0%	0/0%	

AZ7a	0/0%	0/0%	0/0%	0/0%	0/0%	0/0%	0/0%	3+*/50%	0/0%	0/0%	0/0%	0/0%	0/0%	0/0%	0/0%	

AZ7b	0/0%	0/0%	0/0%	0/0%	0/0%	1/11.1%	0/0%	1/16.7%	0/0%	1+*/100%	0/0%	0/0%	0/0%	0/0%	0/0%	

AZ8	0/0%	1+*/50%	1/33.3%	0/0%	0/0%	0/0%	0/0%	0/0%	0/0%	0/0%	0/0%	0/0%	0/0%	0/0%	2+*/100%	

AZ9	0/0%	0/0%	0/0%	0/0%	0/0%	1+*/11.1%	0/0%	0/0%	0/0%	0/0%	0/0%	0/0%	0/0%	0/0%	0/0%	

AZ10	0/0%	0/0%	0/0%	0/0%	0/0%	0/0%	0/0%	0/0%	0/0%	0/0%	0/0%	1+*/100%	0/0%	0/0%	0/0%	

AZ11	0/0%	0/0%	0/0%	0/0%	0/0%	1/11.1%	0/0%	0/0%	3+*/75%	0/0%	0/0%	0/0%	1+*/100%	0/0%	0/0%	

AZ12	0/0%	0/0%	0/0%	0/0%	1/25%	1/11.1%	0/0%	0/0%	0/0%	0/0%	1+*/50%	0/0%	0/0%	0/0%	0/0%	

AZ13	0/0%	0/0%	0/0%	0/0%	0/0%	0/0%	0/0%	0/0%	1+*/25%	0/0%	0/0%	0/0%	0/0%	0/0%	0/0%	

AZ14	0/0%	0/0%	0/0%	0/0%	0/0%	0/0%	0/0%	0/0%	0/0%	0/0%	0/0%	0/0%	0/0%	1+*/50%	0/0%	

AZ16	0/0%	1+*/50%	0/0%	0/0%	0/0%	1/11.1%	0/0%	0/0%	0/0%	0/0%	1+*/50%	0/0%	0/0%	1+*/50%	0/0%	

AZ19	1+*/100%	0/0%	0/0%	1+*/100%	0/0%	0/0%	0/0%	0/0%	0/0%	0/0%	0/0%	0/0%	0/0%	0/0%	0/0%	

*Ball disp.*	GZ2b	GZ3a	GZ3b	GZ3c	GZ4a	GZ4b	GZ5a	GZ5b	GZ5c	GZ6b	GZ6c	GZ7b	GZ10	GZ11	GZ12

AR	0/0%	2/100%	2/66.7%	0/0%	3/75%	7+*/77.8%	3/60%	2/33.3%	1/25%	0/0%	0/0%	0/0%	0/0%	1/50%	0/0%	38.762 0.085 0.684

B	1+*/100%	0/0%	0/0%	1+*/100%	1/25%	0/0%	1/20%	1/16.7%	0/0%	0/0%	0/0%	0/0%	0/0%	0/0%	0/0%	

GR	0/0%	0/0%	1/33.3%	0/0%	0/0%	2/22.2%	1/20%	3/50%	3/75%	1/100%	2/100%	1/100%	1/100%	1/50%	2/100%	

*Nº contac*.	GZ2b	GZ3a	GZ3b	GZ3c	GZ4a	GZ4b	GZ5a	GZ5b	GZ5c	GZ6b	GZ6c	GZ7b	GZ10	GZ11	GZ12	

1C	1/100%	1/50%	1/33.3%	0/0%	4/100%	7/77.8%	4/80%	6/100%	2/50%	0/0%	1/50%	0/0%	1/100%	1/50%	0–*/0%	38.365 0.092 0.682

2C	0/0%	0/0%	**2+** */66.7%	**1+** */100%	0/0%	1/11.1%	1/20%	0/0%	0/0%	**1+** */100%	1/50%	**1+** */100%	0/0%	0/0%	1/50%	

3C+	0/0%	1/50%	0/0%	0/0%	0/0%	1/11.1%	0/0%	0/0%	2+*/50%	0/0%	0/0%	0/0%	0/0%	1/50%	1/50%	

*Shot surf*.	GZ2b	GZ3a	GZ3b	GZ3c	GZ4a	GZ4b	GZ5a	GZ5b	GZ5c	GZ6b	GZ6c	GZ7b	GZ10	GZ11	GZ12

H	0/0%	1/50%	1/33.3%	0/0%	2/50%	5/55.6%	2/40%	1/16.7%	1/25%	0/0%	0/0%	0/0%	0/0%	0/0%	0/0%	9.545 0.795

L	1/100%	1/50%	2/66.7%	1/100%	2/50%	4/44.4%	3/60%	5/83.3%	3/75%	1/100%	2/100%	1/100%	1/100%	2/100%	2/100%	

*Shot oppo.*	GZ2b	GZ3a	GZ3b	GZ3c	GZ4a	GZ4b	GZ5a	GZ5b	GZ5c	GZ6b	GZ6c	GZ7b	GZ10	GZ11	GZ12

OP	0/0%	1/50%	0/0.0%	1/100%	2/50%	5/55.6%	1/20%	3/50%	2/50%	1/100%	1/50%	1/100%	0/0%	0/0%	2/100%	13.660 0.475

NOP	1/100%	1/50%	3/100%	0/0%	2/50%	4/44.4%	4/80%	3/50%	2/50%	0/0%	1/50%	0/0%	1/100%	2/100%	0/0%	

*Goal zone*	GZ2b	GZ3a	GZ3b	GZ3c	GZ4a	GZ4b	GZ5a	GZ5b	GZ5c	GZ6b	GZ6c	GZ7b	GZ10	GZ11	GZ12

PZ1	0/0%	0/0%	1/33.3%	0/0%	1/25%	1/11.1%	1/20%	0/0%	0/0%	0/0%	0/0%	0/0%	0/0%	1/50%	0/0%	78.742 0.222

PZ2	1/100%	0/0%	2/66.7%	0/0%	0/0%	3/33.3%	4/80%	1/16.7%	0/0%	0/0%	2/100%	1/100%	1/100%	0/0%	0/0%	

PZ3	0/0%	0/0%	0/0%	1/100%	0/0%	0/0%	0/0%	1/16.7%	0/0%	0/0%	0/0%	0/0%	0/0%	1/50%	0/0%	

PZ4	0/0%	0/0%	0/0%	0/0%	1/25%	0/0%	0/0%	1/16.7%	0/0%	0/0%	0/0%	0/0%	0/0,%	0/0%	0/0%	

PZ5	0/0%	0/0%	0/0%	0/0%	2/50%	1/11.1%	0/0%	0/0%	2/50%	0/0%	0/0%	0/0%	0/0%	0/0%	1/50%	
PZ6	0/0%	2/100%	0/0%	0/0%	0/0%	4/44.5%	0/0%	3/50%	2/50%	1/100%	0/0%	0/0%	0/0%	0/0%	1/50%	

Note: Table 4 lists the frequencies and their % within the Goalkeeper Location. With +/-* are those values whose adjusted residuals are between z> 1.96 or Z < -1.96 with statistically significant difference *(p* < .05). The “involuntary” actions (assistant from defending team or goal on own goal) were excluded from the analyses (n = 44).

Through the adjusted residues test, it was verified that in certain cases there are significant differences between the actual count and the expected count, with the adjusted residues being normally positive (count higher than expected).

The results of the analysis showed a significant association between the assistant location and the scorer location (p=0.004, ES=0.930). As depicted in Table 4, a high percentage of goals (9 goals; 20.4%) were preceded by: a side pass from the right corridor of the attack (from zone 1 to zone 4b: 3 goals; 6.8%); a back pass from the back line (from zone 7a to zone 5b: 3 goals; 6.8%); and a short pass in the central zone towards the inside of the penalty area (from zone 11 to zone 5c: 3 goals; 6.8%). It should also be noted that 100% of the assists made from zone 7a were finished in zone 5b (see Table 4; greater amount than expected according to the adjustment). The association between the ball disposition during the assist and the location of the scorer was not statistically significant (p=0.085, ES=0.684). Although it did not reach statistical significance, we believe that it is worth highlighting the fact that in zone 4b, seven of the 21 goals (16% of all the total goals analysed) that came from an “aerial” ball were scored.

Finally, a non-significant relationship was also found between the number of scorer contacts and the scorer location (p=0.092, ES=0.682). It should be noted that more than half of the goals scored (54.5%) were scored from the 4 zones that include the projection of the goal to the penalty spot: 4a, 4b, 5a and 5b (Figure 2). Thus, of the 24 goals scored from these zones, 21 of them (47.7% of the total goals) were achieved at the first contact with the ball (Table 4).

## DISCUSSION

The present study examines the goals scored during the UEFA Women’s EURO 2022, with the objective of delineating contextual variables associated with goal occurrences and their influence on match outcomes. Specifically, our focus is on identifying the technical-tactical indicators that characterize goal-scoring patterns. In this novel approach, apart from considering a large number of assist zones to better represent the spatial-situational context, two novel and highly relevant technical-tactical indicators were analysed: the ball disposition during the assist and the origin and shot direction of the goal.

In relation to the *contextual variables*, our results showed an average of goals per match (3.1) higher than in previous championships: Women’s Euro 2017 (2.2), or in the French World Cup finals in 2019 (2.8). It was also observed that 2% more goals were scored in the second half than in the first. Although the scoring difference is very small, these data are in line with those obtained in previous world tournaments (2012 Olympic Games [17]; World Cup 2019 [21]).

The goals scored from dynamic attacks (65.9%) and set-pieces (34.1%) were similar to those observed in the 2019 Women’s World Cup (61.1% and 38.9% respectively [22]; data registration from the official FIFA website) and in the Spanish Women’s National League [18]. Also, in line with previous studies, it was confirmed that scoring first markedly increased the probability of winning the match [20, 31, 32]. In our study, only one team was able to come back from a match during regulation time. In the present study, 14 goals (14.7%) were scored during the first 15 minutes of the tournament, of which 10 served to advance on the scoreboard. These data highlight the importance of this time period in the development of the match and its final outcome in the championship.

As for the results based on the technical-tactical variables, regarding the analysis of the assistant location, it stands out that in up to 20 goals the assist was given from the lateral corridors outside the penalty area (zones 1 and 8 in Figure 1, 23.2% of the intentional assists). However, it should be considered that half of the assists (10) came from corners or free kicks. Regarding this aspect, it is also noteworthy that 17 assists originated from the flanks of the penalty area (zones 2a: 2b and 7a: 7b with 19.7% of intentional assists, Figure 1). Thus, the overall assistance percentage from all these lateral zones is 42.9%. It rises to over 50% if the central corridor (Z11) is considered. This result aligns with the conclusions offered by Espada et al. [23], who analysed only the 29 goals of a women’s team in the Portuguese league. Other previous studies have also highlighted the importance of crosses in scoring goals in women’s football [19, 33].

It is noteworthy, from the training perspective, that during the dynamic attack phase, the strategy of delivering crosses from the flanks is often employed by coaches, especially when the opposing team maintains a compact defence or is positioned narrowly. Note that in our study there were only three cases in which the assist came from a player who was in her own field. These data are in line with the results obtained in previous studies which verified that more goals were scored using short passes than long ones [24]. For instance, Mara et al. [33] reported that 51% of the goals came from passes between 15 and 35 m, a situation that was also predominant in the 2022 Women’s Euro. These results may be closely related to one of the most significant findings of our study, linked to the analysis of the situations in which the ball reached the scorer. Our results have shown that in more than half of the goals the shot was made after an aerial ball (51.2%), with the leg (66.3%) and as result of a header (32.5%). In this regard, it is also interesting to highlight the increase in heading goals compared to those scored in the 2019 World Cup, 20% [21], and in the Spanish League, 11.8% [18]. However, it is important to consider the higher number of goals analysed in these studies (144 and 330 respectively). In our case, these patterns of results might be linked to the zones of higher prevalence (corners) and the most common type of assist (aerial).

Our study also showed that a large percentage of the voluntary goals were scored from the zone that extends from the goal area to the edge of the penalty area (83.1%; all zones including from Z3 to Z6). The high number of goals scored inside the penalty area (72 voluntary and 4 involuntary) conditioned the number of contacts that the finishers used to score the goal, as the majority of goals (70%) were achieved on the first contact, similar to what happened during the 2019 World Cup [21, 22]. This seems logical considering, as we have seen, that most goals are scored near the goal, which usually implies high space-time pressure to finish the play. Unsurprisingly, the number of goals scored from outside the penalty area was considerably low (9.1% of the total). This pattern of data (8 goals; 5 from Z11) is consistent with other studies that utilized a similar zone division [18, 22], although in both cases the percentage was around 15%. Despite Espada et al. [23] using different zones for scoring locations, a similar trend was observed, with goals from outside the penalty area accounting for 17% in their study.

Another relevant finding observed in our results is the high percentage (51.2%) of goals scored without direct opposition to the goal scorer at the time of the shot. In comparison, Kubayi [24] reported that from the 17.1% of shots that ended in a goal in the 2019 World Cup, on 6.7% of the occasions the shooter had direct defender pressure (which represents about 40%). The present observation of a high number of unopposed goals may have been related to the type of attack, a variable we did not analyse in the study.

Regarding the shot direction and the goal penetration zone, it is notable that almost 60% of the goals entered at the first post or through the centre of the goal. The shot direction was towards the low zones of the goal in 70.5% of cases (PZ2, PZ4 and PZ6), a fact that could be highly influenced by the shot distance (up to 11 m). Díaz-Serradilla et al. [18] observed that the majority of shots from the central zones of the penalty area entered the low zones of the goal, with a tendency to cross shots from the sides, and look for the upper zones of the goal in shots from outside the penalty area. These types of analyses are of high interest especially for goalkeepers, since many of those patterns can be reproduced in specific training sessions with the aim of analysing their positioning in relation to the goal, the body position in the shot or the type of reaction. For instance, in a cross, optimal positioning is crucial, requiring consideration of the assistant’s location, the scorer, and the goal. Therefore, mastering the ability to diversify attention whenever feasible is essential.

The present study provided a novel analysis regarding the origin of the ball in the shot, also taking into consideration the opponents who contacted the ball after the pass action, and before the scorer. It was observed that in almost 21% of the goals, the ball came from a defending player, leaving the ball in most situations in the zone in front of the goal, and at a reduced distance to the goal line (less than 11 m). These results must highlight the importance of “second balls” (loss or rejection) in the vicinity of the goal, seeking to develop a more active than reactive tactical intention when looking for or avoiding goals [34].

The analysis of associations between technical-tactical indicators related to the assistant location and scorer location is highly innovative. The results of the bivariate analysis revealed a significant correlation between the location of the assist and the goal scorer. Specifically, there was a greater frequency of goals in the central zones of the penalty area when the ball came from a lateral pass (Z1 to Z4b and from Z7a to Z5b), or a short pass in the central corridor toward the inside of the penalty area (Z11 to Z5c).

This may be because in this type of situation both the goalkeeper and her teammates show a great concern to defend the zones closest to the goal, neglecting others (periphery of the goal area).

Some authors have stated that, “in general terms, the offensive process in women’s football is clearly ineffective” [35]. This opinion, together with the scarcity of research that analyses in depth the context of the assistant and the shooter, as well as the potential dependency relationships that might be established between them, justifies the need to continue investigating the goal scoring patterns in women’s football. In this regard, the present study significantly enhances our understanding of goal patterns in elite women’s football.

Our findings offer valuable insights that can inform coaches in designing more effective training practices tailored to the observed scoring patterns in elite women’s football. Specifically, coaches can utilize our results as a reference framework for creating situational training tasks for goalkeepers, defenders, and attackers that align with the most common scoring situations identified in our study.

Furthermore, coaches can use our findings to inform their selection of offensive and defensive strategies in competition, taking into account factors such as the most recurrent assisting and shooting zones, the number of scorer contacts, the ball disposition and the direction of the ball depending on the assist.

By integrating these insights into their training practices and strategic planning, coaches can better prepare their teams to capitalize on scoring opportunities while minimizing defensive vulnerabilities. We believe that our research contributes valuable information to the optimization of performance strategies in elite women’s football.

## Limitations of the study

Despite the novelty of our methodological approach and the interest of the results obtained, which can be used as a reference in future studies, we now present certain limitations that should be considered in the interpretation of the results.

The primary limitation of this study is linked to the sample size (n = 95). This constraint is virtually unavoidable, given that the research objective revolves around analysing goals in a competition with a short duration (31 matches comprising the final phase of the UEFA Women’s EURO 2022). It is also important to consider that our approach did not take into account the non-scoring attacking attempts, hindering the interpretation of the effectiveness of the actions. Additionally, differences in the competitive level of teams were not taken into account, with all the implications that this entails.

In connection with the development of the *ad hoc* instrument and the reliability of the data, there are several noteworthy aspects. Regarding the instrument, it is necessary to point out that certain criteria restrict the context in which the actions are developed, despite having defined different categories. For example, in the analysis of the location of the assistant and the scorer the moments prior to the assist or the shot were not taken into consideration, but only the location at that precise moment. This fact implies the absence of highly valuable information for the analysis (e.g., the place of initiation of the movement of the shooter).

It is also worth noting that in some goals, the precise assistant location and scorer location has proved complex given the high number of zones included in the fieldgram and the plane offered by some of the video providers. This drawback has been resolved by contrasting video footage with the generic references offered by the reports of Wyscout (for assists) and InStat platforms (for shots).

Regarding the reliability of the recording, it is worth noting that it was conducted by a single observer, and therefore, although a very successful intra-observer check was carried out, no inter-observer analysis was possible. In the event that future studies along these lines would want to use the same instrument, an inter-observer reliability study would be necessary.

Finally, it is important to emphasize that the small sample size and the high number of finishing zones might affect the statistical power of our analyses. This is the main reason that our analysis exploring the association of the scoring zone with other variables showed that the expected values in each cell were not representative in some cases.

Furthermore, the intricate nature of goal-scoring opportunities suggests potential interactions among independent variables, highlighting the significance of employing a multivariate analysis approach. However, it is important to acknowledge that this study represents an initial exploration into the analysis of goals and goal-related actions. Future research should prioritize conducting multivariate and probabilistic analyses to provide deeper insights into the complexities of the subject matter.

## CONCLUSIONS

In this study, goals from the UEFA Women’s EURO 2022 were analysed to identify technical-tactical indicators related to goal scoring. We adopted an innovative approach by examining variables that, to our knowledge, had not been previously studied, such as the ball disposition during the assist and the origin and shot direction on goal.

Our findings revealed that a high percentage of goal assists originated from the sides near the corners, with the most frequent shooting zones extending from the goal line to the penalty spot. Additionally, it is noteworthy that half of the goals resulted from an aerial ball, with the leg and first-touch finishing being the most common situations.

The present study is also highly innovative in pointing out the significant association between technical-tactical indicators related to the assistance zones and shooting. The importance of these data in studying goal patterns holds great value for future research in this field.
